# The interplay between viral molecular mimicry and host chromatin dynamics

**DOI:** 10.1080/19491034.2023.2216560

**Published:** 2023-05-22

**Authors:** Shumin Xiao, Yajing Wang, Shan Shan, Zheng Zhou

**Affiliations:** a National Laboratory of Biomacromolecules, CAS Center for Excellence in Biomacromolecules, Institute of Biophysics, Chinese Academy of Sciences, Beijing, China; b University of Chinese Academy of Sciences, Beijing, China

**Keywords:** Viruse, molecular mimicry, phase separation, chromatin, histone

## Abstract

Molecular mimicry is a commonly used mechanism by viruses to manipulate host cellular machinery and coordinate their life cycles. While histone mimicry is well studied, viruses also employ other mimicry strategies to affect chromatin dynamics. However, the relationship between viral molecular mimicry and host chromatin regulation is not well understood. This review summarizes recent advances in histone mimicry and explores how viral molecular mimicry influences chromatin dynamics. We also discuss how viral proteins interact with both intact and partially unfolded nucleosomes and compare the distinct mechanisms governing chromatin tethering. Finally, we address the role of viral molecular mimicry in regulating chromatin dynamics. This review provides new insights into viral molecular mimicry and its impact on host chromatin dynamics, paving the way for the development of novel antiviral strategies.

## Introduction

The nucleosome is a fundamental unit of chromatin that consists of variable histone content, DNA sequences, and epigenetic markers. It adopts a disc-like structure with a diameter of ~11 nm and a thickness of ~5.5 nm. The 147 bp DNA is wrapped around the core histone octamer, which contains four types of core histones (H2A, H2B, H3, and H4) in the form of a left-hand helix about 1.65 turns [1]. Nucleosomes are packaged and folded into 30 nm chromatin fibers under the action of the linking histone [2], and further condensed into higher-order structures [3,4]. The tight packing of DNA during chromatin condensation prevents the binding of the transcriptional machinery, leading to gene silencing. Conversely, relaxed chromatin allows transcriptional machinery to access DNA, thereby ensuring gene activation. Chromatin undergoes dynamic changes during the entire cell cycle, which are regulated by several key players, including DNA methylation, histone modification, histone variant, chromatin remodeling, and noncoding RNA [5–7].

Molecular mimicry is a common mechanism used by pathogens, such as viruses, to interfere with host surveillance systems, facilitate viral infections, and promote pathogen survival. Despite its classical role in autoimmune reactions, molecular mimicry manipulates host chromatin dynamics to influence the host surveillance system. In this review, we summarize the current understanding of the viral molecular mimicry strategy used by viruses and discuss its effects on chromatin dynamics. We discuss recent findings of viral proteins using mimicry strategy and catalog several mechanisms by which viral proteins interact with intact and partially unfolded nucleosomes. Finally, we discuss the recent findings of viral proteins regulating chromatin phase separation.

## Main

Viruses use molecular mimicry, which relies on the structural similarity between host and viral proteins, to regulate chromatin dynamics. The strategy of viral molecular mimicry can be categorized by type of host proteins, such as histones, nucleosome-binding proteins, histone chaperones, and proteins undergoing phase separation, which are exploited by viruses for molecular mimicry (Table 1). It has been demonstrated that the viral histone mimicry influences host epigenetic processes and enables phase separation, thereby impacting chromatin-mediated control of gene expression. These topics have been thoroughly reviewed elsewhere [8,9].

**Table 1.** Mechanism and function of viral molecular mimicry.

Type of host protein	Type of virus	Viral protein	Function of viral molecular mimicry
Histone	CpBV	CpBV-H4	Inhibit gene expression
	Marseillevirus	Doublet histones	Protect viral genome
	H3N2	NS1	Facilitate transcription or RNA splicing
	HDV	S-HDAg	Facilitate replication and chromatin remodeler recruitment
	Ad	VII	Inhibit H2A.X accumulation
	SARS-CoV-2	ORF8	Lower the DNA accessibility
Nucleosome-binding protein	KSHV	LANA	Chromatin compaction
	CMV	IE1	Chromatin decompaction
	SPV	Gag	Chromatin tethering
Histone chaperone	EBV	BKRF4	Nucleosomal DNA unwrapping
	Whispovirus	ICP11	Interfere with nucleosome assembly, inhibits DNA damage response signaling
Protein undergoing phase separation	EBV	EBNA2/EBNALP	Promote chromatin decompation and acetylation, regulates latent viral transcription
	γ/α herpesvirus	ORF52/VP22	Immune evasion
	HIV-1	LTR	Enhance the efficiency of gene regulation
	SARS-CoV-2	N protein	Suppress the innate antiviral immune response

## Viral histone and mechanisms of histone mimicry

**Figure 1.**
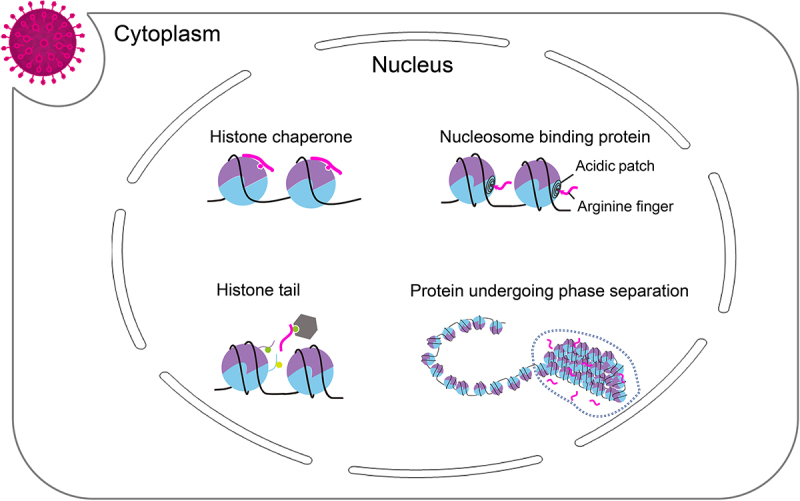
Model of different ways of viral molecular mimicry for host infection.

Many viruses employ histone mimicry strategies to compact their genomes (Figure 1). Nuclear DNA viruses and retroviruses organize their genome into nucleosomes through eukaryotic histones, while others encode their own histone-like proteins [10]. Virus-encoded histone-like proteins are structurally distinct from eukaryotic histones, suggesting specialized functions [11]. For example, the insect polydnavirus CpBV encodes an orthologue of the insect histone H4, which can be incorporated into host nucleosomes to inhibit genes expression [12]. A recent study of Marseillevirus revealed two doublet histones homologous to the four core histones in eukaryotes, forming a tetrameric nucleosome containing only 121 bp DNA [13,14], which closely resembles a partially unfolded canonical nucleosome structure and is implicated in viral genome protection [15].

In addition to histone mimicry, many viral proteins show remarkable sequence similarity to specific motifs located in the N-terminal tail of histone H3, which are sites of multiple post-translational modifications [16]. For instance, the Non-structural protein 1 (NS1) of influenza A virus subtype H3N2 contains a carboxy-terminal ARSK motif [17,18], which is recognized by the polymerase-associated factor 1 complex PAF1C and chromatin remodeler Chd1 to facilitate virus transcription and mRNA splicing [19–23]. Similarly, the hepatitis D virus (HDV) encodes a small HDV antigen (S-HDAg) containing the KXXR motif, which is recognized by BAZ2B, a regulatory subunit of in the chromatin remolding ISWI and RNA Pol II to fulfill HDV’s replication [21]. Furthermore, Protein VII in human adenoviruses has a H3 ARSK-like sequences AKKRS [24]. The acetylation of this motif in the ectopically expressed Protein VII has been linked to chromatin organization [25]. A recent study found that the SARS-CoV-2 protein ORF8, which contains an ARKS motif targeted by H3K9 acetyltransferase KAT2A (i.e., human GCN5), decreases DNA accessibility. This effect was observed in infected cells, which displayed increased levels of repression markers (H3K9me3 and H3K27me3) and decreased levels of activation markers (H3K27ac). The histone mimicry mechanism in SARS-CoV-2 also appears to play a role in viral transcription since depletion of the ORF8 ARKS motif reduces the copy number of the viral genome [26].

## Nucleosome anchoring: viral proteins targeting the acidic patch

Viral proteins use various strategies to tether themselves to chromatin, including mimicry of chromatin-binding proteins. Many chromatin-binding proteins interact with the acidic patch on the nucleosome surface region selectively via an arginine finger motif, which is also used by many nonhistone proteins (Figure 1) [27–29]. Among all viral proteins known to interact with nucleosomes, LANA in Kaposi sarcoma-associated herpesvirus (KSHV), IE1 of cytomegalovirus (CMV), and Gag of spumaretrovirus (SPV) interact with intact nucleosomes via arginine finger motif [30–34]. In addition to the acidic patch, viruses employ other components of the nucleosome, including the histone octamer and nucleosomal DNA, for chromatin tethering [28,35]. For example, Adenovirus (Ad) protein VII is a highly basic protein that binds to viral DNA to form a ‘bead-like structure’ similar to nucleosomes [36]. Protein VII also binds to the nucleosome at the linker DNA region to inhibit H2A× accumulation and host DNA damage response [37]. Furthermore, Protein VII recruits high mobility group box (HMGB) proteins to alter the higher-order structure of chromatin, leading to obstruction of cell cycle progression [38]. These findings underscore the critical role of chromatin tethering in ensuring the association of viruses with chromatin and in affecting the chromatin dynamic [39].

## Histone chaperones mimicry: viral proteins and nucleosome assembly regulation

Interactions between the arginine finger and acidic patch are primarily observed in intact nucleosomes. However, recent research on BKRF4 from Epstein–Barr virus (EBV) has demonstrated that BKRF4 can also bind to partially unfolded nucleosomes [40]. Interestingly, BKRF4 interacts with the H2A-H2B dimer via a ‘triple-anchor’ binding mode, inducing the unwrapping of nucleosome DNA and exposing histone octamer-binding sites typically concealed. Therefore, BKRF4 tends to interact with partially unfolded nucleosomes at DNA breaks, interfering with the recruitment of RNF168 and blocking the propagation of DDR signals [41]. The binding mode between BKRF4 and histone H2A-H2B or H3-H4 is reminiscent of the histone chaperone property of BKRF4, suggesting that BKRF4 adopts a histone chaperone mimicry mechanism to interfere with nucleosome assembly and downstream protein recruitment [40]. Similarly, the white spot syndrome virus protein ICP11, referred to as DNA mimicry, interacts with histone H2A, H2B, H3, and H2A.X, likely interfering with nucleosome assembly and inhibiting DNA damage response signaling at DNA double-strand breaks [42]. It is conceivable that the DNA mimicry proteins function as histone chaperones, given that they both occupy the DNA binding site on the target proteins to eliminate DNA binding (Figure 1).

## Impact of virus-host protein interactions on phase separation dynamics

Recent studies have shown that chromatin dynamics are influenced by a phenomenon called chromatin liquid–liquid phase separation (LLPS) (Figure 1). It has been reported that the histone tail drives the process of chromatin phase separation, which is counteracted by histone acetylation [43]. Furthermore, the activation domains of transcription factors can form phase separation condensates [44]. There is emerging evidence that viruses may exploit LLPS for chromatin reorganization. For instance, the Epstein–Barr virus (EBV) proteins EBNA2 and EBNALP utilize the LLPS to form nuclear condensates that drive the transcription of viral genes [45]. The LLPS of EBNA2 reorganizes chromatin topology and recruits the histone acetyltransferase p300 to promote histone H3K27 acetylation [46]. The sequence similarity between EBNA2/EBNALP and transcription factors implies a mimicry strategy that could govern LLPS, adding another facet of chromatin regulation targeted by viral molecular mimicry [45].

It is important to note that LLPS of viral proteins not only impacts host chromatin structure but also enhances viral fitness. For instance, herpesvirus tegument proteins ORF52/VP22 form condensates with DNA through LLPS [47]. The accumulation of ORF52/VP22 effectively disrupts pre-formed cGAS-DNA condensation and prevents cGAS detection of the presence of DNA [47,48]. At the HIV-1 long terminal repeat (LTR), histone chaperone CAF-1 (chromatin assembly factor 1) is enriched and forms nuclear bodies with phase separation properties to facilitate HIV-1 latency establishment [49]. Similarly, SARS-CoV-2 nucleocapsid protein (NP) undergoes LLPS upon binding to viral RNA, leading to a virus-triggered NF-κB signaling activation [50]. The LLPS of NP suppresses the innate antiviral immune response by inhibiting the Lys63-linked poly-ubiquitination and activity of MAVS [51].

## Discussion

Extensive evidence has demonstrated that viruses have evolved numerous strategies, beyond histone mimicry, to influence chromatin dynamics. Extensive evidence has shown that viruses have evolved a variety of strategies, beyond histone mimicry, to influence chromatin dynamics. This review discusses other mimicry strategies employed by viruses, such as mimicking nucleosome-binding proteins, histone chaperones, and proteins undergoing phase separation. The remarkable diversity of mimicry strategies suggests that not all mechanisms used by viruses to modulate chromatin structure have been fully characterized. Indeed, recent studies have shown that various viruses produce factors to regulate the 3D organization of the host genome, and they exploit the altered chromatin architecture to promote viral fitness [52]. Previous studies have underscored the significance of intrinsically disordered regions (IDRs) in viral protein mimicry [9]. IDRs, characterized by their low complexity and high flexibility, are exploited by viruses to establish weak multivalent interactions that underlie the dynamic nature of chromatin. Understanding the mechanisms by which IDRs govern dynamic interactions is crucial. This knowledge could facilitate the identification of functional IDPs in viruses. Furthermore, the viral mimicry strategy could inspire the design of novel proteins that could have therapeutic benefits.
